# Development of a multisensor biologging collar and analytical techniques to describe high‐resolution spatial behavior in free‐ranging terrestrial mammals

**DOI:** 10.1002/ece3.70264

**Published:** 2024-09-23

**Authors:** Michael S. Painter, Václav Silovský, Justin Blanco, Mark Holton, Monika Faltusová, Rory Wilson, Luca Börger, Liza Psotta, Fabian Ramos‐Almodovar, Luis Estrada, Lukas Landler, Pascal Malkemper, Vlastimil Hart, Miloš Ježek

**Affiliations:** ^1^ Department of Biology Barry University Miami Shores Florida USA; ^2^ Department of Game Management and Wildlife Biology, Faculty of Forestry and Wood Sciences Czech University of Life Sciences Prague Czech Republic; ^3^ Electrical and Computer Engineering Department United States Naval Academy Annapolis Maryland USA; ^4^ Swansea Lab for Animal Movement, Biosciences College of Science, Swansea University Swansea UK; ^5^ Department of Music Education Folkwang University of the Arts Essen Germany; ^6^ Department of General Zoology, Faculty of Biology University of, Duisburg‐Essen Essen Germany; ^7^ Genomics and Computational Biology Graduate Group, Perelman School of Medicine University of Pennsylvania Philadelphia Pennsylvania USA; ^8^ Department of Psychology University of Miami Coral Gables Florida USA; ^9^ Institute of Zoology University of Natural Resources and Life Sciences Vienna Austria; ^10^ Research Group Neurobiology of Magnetoreception Max Planck Institute for Neurobiology of Behavior – caesar Bonn Germany

**Keywords:** accelerometer, behavioral classification, biologging, dead‐reckoning, GPS, machine learning, magnetic compass heading, magnetometer

## Abstract

Biologging has proven to be a powerful approach to investigate diverse questions related to movement ecology across a range of spatiotemporal scales and increasingly relies on multidisciplinary expertise. However, the variety of animal‐borne equipment, coupled with little consensus regarding analytical approaches to interpret large, complex data sets presents challenges and makes comparison between studies and study species difficult. Here, we present a combined hardware and analytical approach for standardizing the collection, analysis, and interpretation of multisensor biologging data. Here, we present (i) a custom‐designed integrated multisensor collar (IMSC), which was field tested on 71 free‐ranging wild boar (*Sus scrofa*) over 2 years; (ii) a machine learning behavioral classifier capable of identifying six behaviors in free‐roaming boar, validated across individuals equipped with differing collar designs; and (iii) laboratory and field‐based calibration and accuracy assessments of animal magnetic heading measurements derived from raw magnetometer data. The IMSC capacity and durability exceeded expectations, with a 94% collar recovery rate and a 75% cumulative data recording success rate, with a maximum logging duration of 421 days. The behavioral classifier had an overall accuracy of 85% in identifying the six behavioral classes when tested on multiple collar designs and improved to 90% when tested on data exclusively from the IMSC. Both laboratory and field tests of magnetic compass headings were in precise agreement with expectations, with overall median magnetic headings deviating from ground truth observations by 1.7° and 0°, respectively. Although multisensor equipment and sophisticated analyses are now commonplace in biologging studies, the IMSC hardware and analytical framework presented here provide a valuable tool for biologging researchers and will facilitate standardization of biologging data across studies. In addition, we highlight the potential of additional analyses available using this framework that can be adapted for use in future studies on terrestrial mammals.

## Graphical abstract

Here we present the development and extensive field testing of a multisensor biologging collar in wild boar (*Sus scrofa*), coupled with classification techniques for identifying boar behavior from raw accelerometer data and, for the first time, provide a detailed characterization of magnetic heading data derived from raw magnetometer data in free‐roaming individuals. By integrating our behavioral classifier and magnetic orientation data, we also explore dead‐reckoning path reconstruction techniques that can provide a wealth of additional information for studies of animal movement and behavior.

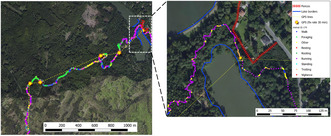

## 1 INTRODUCTION

In recent decades, animal‐borne sensors designed to monitor physiology, behavior, movement, and environmental conditions have revolutionized studies of animal ecology in diverse taxa across a range of spatiotemporal scales (Ropert‐Coudert & Wilson, 2005; Rutz & Hays, 2009; Williams et al., 2020; Wilmers et al., 2015). This has been made possible due to advances in sensor technology, data management, and analytical techniques, which now underpin both theoretical and applied research on wild animals (Cooke et al., 2012; Rattenborg et al., 2016; Vyssotski et al., 2006; Wilmers et al., 2015). However, the emergence of novel biologging techniques requires a multidisciplinary approach, often relying on diverse expertise in areas beyond wildlife ecology (Jolles, 2021; Kays et al., 2022; Portugal & White, 2018; Tuia et al., 2022; Wild et al., 2023). Furthermore, animal‐borne electronics and data sets are increasingly tailored to a particular study or research group, making access to, and comparison between, biologging studies challenging.

Triaxial accelerometers and magnetometers form the bedrock of biologging studies and are capable of providing high‐resolution data on animal movement and orientation (Shepard et al., 2008; Williams et al., 2017; Wilson et al., 2008; Yoda et al., 1999). However, transforming and interpreting the often large and complex data sets generated from biologgers into behaviorally and ecologically relevant information requires expertise from disciplines beyond ethology. For example, recent studies have applied various machine learning techniques to identify behaviors from raw accelerometer and/or magnetometer profiles (Balasso et al., 2023; Bidder et al., 2014; Chang et al., 2022; Dentinger et al., 2022; Painter et al., 2016; Studd et al., 2019; Wang, 2019; Yu et al., 2021), in addition to alternative approaches, such as template matching (Walker et al., 2015) and user‐defined algorithms for behavior (Wilson et al., 2018). The performance of such models varies due to factors such as the frequency at which data are recorded and the degree of behavioral variation *within* and *between* the behavioral classes attempting to be identified. To date, no consensus has been reached on a single behavioral classification technique across biologging studies, further hindering comparison between studies and species (Wang, 2019; Wilson et al., 2018; Yu et al., 2021).

Magnetometer data, used in conjunction with accelerometers, can enhance machine learning performance by providing additional information regarding animal body or limb orientation (Alex Shorter et al., 2017; Brewster et al., 2021; Dickinson et al., 2021; Sakai et al., 2019; Williams et al., 2020). In some contexts, triaxial magnetometer data alone have been used to successfully identify behavior in free‐roaming animals (Chakravarty et al., 2019; Williams et al., 2017). In addition, triaxial magnetometers are well suited to provide magnetic heading orientation (Matsumura et al., 2011), although extracting compass headings from raw data is not trivial and depends on sensor calibrations and accelerometer‐based tilt‐compensation corrections (Bidder et al., 2015). Unsurprisingly, calibration techniques are now commonplace in studies that report magnetic heading measurements derived from raw magnetometer data (Fannjiang et al., 2019; Gutzler & Watson III, 2022; Logan et al., 2023; Martín López et al., 2016; Noda et al., 2014); however, few (Wilson et al., 2007) have provided ground truth validation of magnetic compass accuracy and reliability across ecologically realistic movement dynamics or behaviors.

Integration of GPS technology with accelerometer and magnetometer data has further enhanced the accuracy and depth of spatial information in animal tracking studies and is reflected in the widespread deployment of GPS technology across a range of animal studies over the past three decades (Hebblewhite & Haydon, 2010; Katzner & Arlettaz, 2020; Kays et al., 2015). Beyond its utility in providing reliable positional fixes, GPS is now used to improve the performance (e.g., mitigate drift and heading error) of dead‐reckoning path reconstruction that relies on vector integration obtained from synchronized accelerometer and magnetometer data (Gunner et al., 2021) and further underscores the importance of assessing the accuracy of magnetic heading measurements obtained from raw data. Engineering multisensor collars (e.g., GPS, accelerometers, magnetometers) capable of recording and storing large volumes of data over months or years that comply with animal welfare standards remains an additional challenge in biologging research (Cook et al., 2017; Holton et al., 2021; Kenward, 2000; Wilson et al., 1986, 2021).

Here we present the development of a multisensor biologging collar equipped with GPS and triaxial accelerometer and magnetometer sensors that has been extensively tested in free‐ranging wild boar (*Sus scrofa*). In tandem, we have developed a method for classifying ecologically relevant behaviors from raw accelerometer data in wild boar using machine learning techniques and provide a detailed assessment of magnetic compass performance based on raw magnetometer data across a range of behavioral contexts. Our findings suggest that both the collars and analytical techniques are robust, adaptable, and suitable for long‐term studies with terrestrial mammals, and we discuss the broader applications of this work for future wildlife research.

## 2 METHODS

### 2.1 Study site and subjects

Field testing of the integrated multisensor collars (IMSCs) was carried out in unrestricted, natural habitats throughout the Czech Republic. Boar were captured in corral traps, sedated using methods described below (see also Appendix S1), and fitted with the IMSC, then released into the surrounding environment. All data used to develop the behavioral classifier and evaluate magnetic compass performance were collected at a wildlife reserve (49°57′52.7″ N 14°50′14.7″ E) owned by the Czech University of Life Sciences. Inside the reserve, a semi‐natural enclosure (~38 m × ~ 46 m), made from nonmagnetic wood fencing was used to collect ground truth behavioral data (hereafter “behavioral enclosure”) from six adult wild boars between October 2017 and December 2018 (Figure S1). Boar were captured opportunistically using dart tranquilizer methods (see Appendix S1), then were transported inside the behavioral enclosure and fitted with one of the two biologging collar designs (see below). Four infrared game cameras (UOVision UM 565) were installed within the enclosure (Figure S1) to record ground truth data used for behavioral classifier and magnetic heading analyses (see below).

Trapping, handling, and collaring protocols were performed in accordance with the Ethics Committee of the Ministry of the Environment of the Czech Republic number MZP/2019/630/361 and following ARRIVE guidelines (Percie du Sert et al., 2020). See Appendix S1 for additional study site information.

### 2.2 Biologging collar development

**FIGURE 1:**
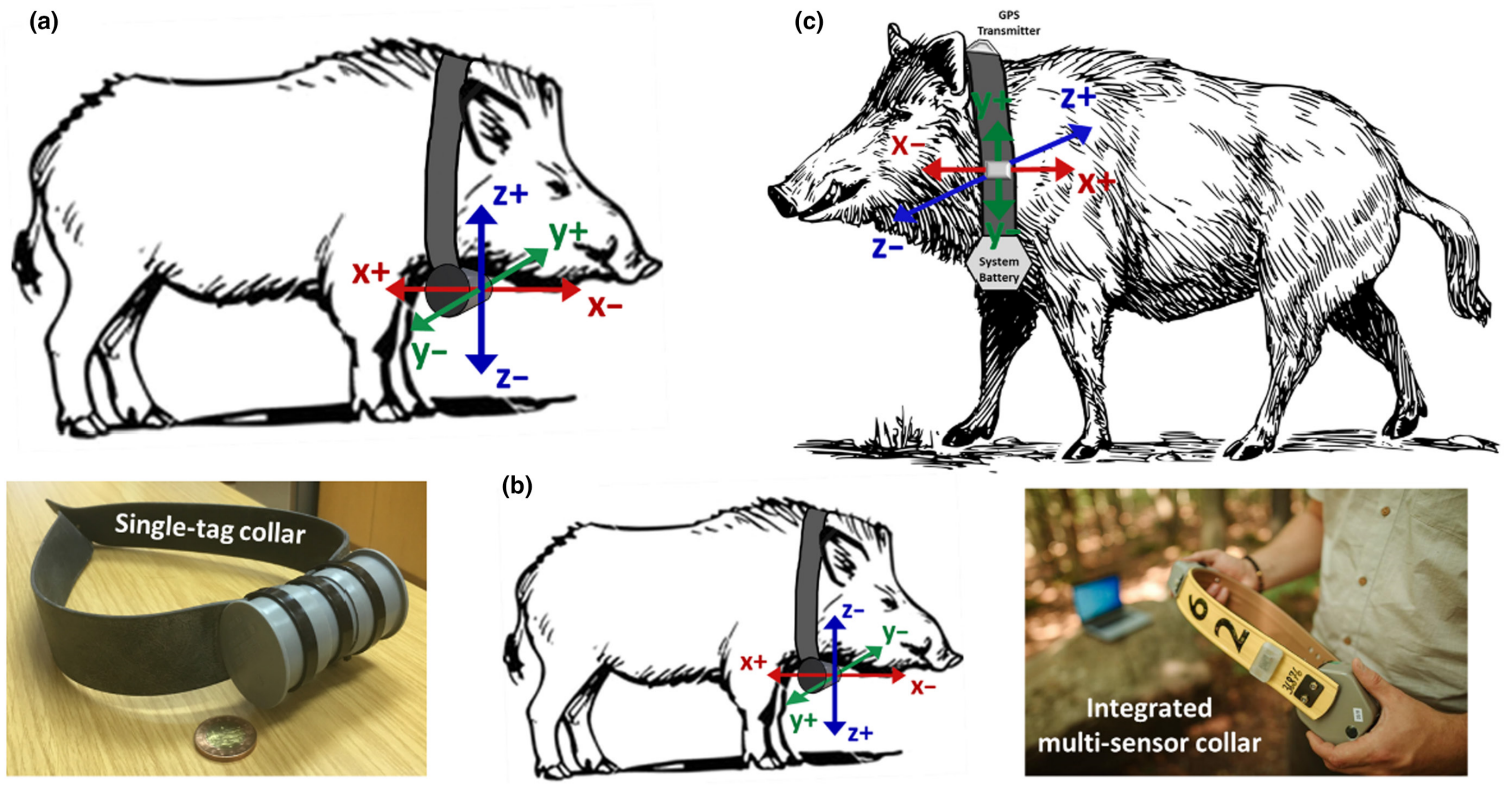
Biologging collars used throughout the study. Accelerometer axes orientation is superimposed on the logger and axis polarity indicates the acceleration value as the axis is pointed toward gravity. Note the different axis alignments between STC designs (a, b). Both the logger position and logger orientation used in all IMSCs (c) differ from the STC logger position and orientations. Photographs of both collar designs are shown below their respective schematics. IMSC, integrated multisensor collars; STC, single‐tag collars.

Two collar systems were designed in this study: “IMSCs” and “single‐tag collars” (STCs), both fitted with Wildbyte Technologies Daily Diary data loggers (http://www.wildbytetechnologies.com/). Loggers were equipped with triaxial accelerometers and triaxial magnetometers (LSM303DLHC, ST Microelectronics) programmed to record continuously at a sample rate of 10 Hz across all six sensors aligned along three orthogonal axes corresponding to the major axes of the boars' bodies (Figure 1).

### 2.3 Integrated multi‐sensor collar (IMSC)

The IMSCs included a “Thumb” Daily Diary tag (18 × 14 × 5 mm) with triaxial accelerometer and magnetometer sensors (LSM9DS1, ST Microelectronics), as well as a Vertex Plus GPS collar, scheduled to record GPS fixes at 30‐min intervals. All accelerometer and magnetometer data was recorded and stored on a removable 32 GB MicroSD card. Collars were equipped with an integrated “drop‐off mechanism” and VHF beacon to enable collar recovery from the field. All collar electronics were powered from a single battery pack (4‐D cell) and the total deployment weight was 716 g. The Daily Diary tag was protected by a custom‐designed polyurethane housing (40 mm × 25 mm × 12 mm) positioned on the outside of the plastic collar belt. The orientation of the tag relative to the collar, as well as the orientation of collar relative to the animal, remained fixed for all IMSC deployments (Figure 1, Table 1).

### 2.4 Single‐tag collars (STCs)

All STCs were equipped with the “Square” Daily Diary tag (27 × 26 × 10 mm) and recorded data to a removable 32 GB MicroSD card. The logger was powered with a single cell 3.6 V lithium battery (SAFT, LS17500CNR) and was oriented and leveled within a 12 cm × 4.8 cm dia PVC‐U cylindrical tube housing secured to a plastic collar belt. Total STC weight was 250 g. All STC housings were positioned ventrally at the base of the animal's neck (Figure 1a,b). However, logger orientation was rotated in one STC deployment (Figure 1b) to test the positional robustness of the behavioral classifier (see below).

See Appendix S1 for additional information regarding collar specifications and deployments.

**TABLE 1:** Collar design and data collection. Overview of collar design, biologger position and orientation, and data type collected (i.e., behavioral classifier training, testing, magnetic heading evaluation), per individual.

Collar design and data collection						
Boar ID	Collar design	Tag position	Tag orientation	Classifier training	Classifier testing	Magnetometer testing
B3	STC	1	A	Yes	No	No
B4	STC	1	B	No	Yes	No
B5	STC	1	A	Yes	No	No
B6	STC	1	A	Yes	No	Yes
B7	STC	1	A	No	Yes	Yes
B30	IMSC	2	C	No	Yes	Yes

*Note*: Numbers and letters listed for Tag Position and Orientation are arbitrary and indicate similarities and differences between collar designs.

Abbreviations: IMSC, integrated multisensor collar; STC, single‐tag collar.

### 2.5 Data collection

Field testing of the IMSC involved 71 collar deployments over a 2‐year period on adult (>12 months, >40 kg) free‐roaming wild boar (52 females, 18 males, 1 unidentified). Collars were evaluated for robustness, capacity, and functionality over 6001 tracking days, cumulatively across all deployments.

Behavioral classifier and magnetic compass performance data were collected from six free‐roaming individuals inside the behavioral enclosure. Before collaring, calibration data used for hard‐ and soft‐iron magnetometer corrections (Gunner et al., 2021; Williams et al., 2017) were collected by rotating the collars through three‐dimensional space for 5 min within the immediate area of the behavioral enclosure. The resulting accelerometer “calibration signature” was also used to time‐sync biologging data with ground truth recordings from each game camera. Upon data retrieval, raw data files were uploaded to DDMT software (Wildbyte Technologies – Swansea University, Singleton Park, Swansea, UK, SA2 8PP), for further processing, including magnetometer calibrations. A summary of data collection and performance evaluations for each collar design is provided in Table 1.

### 2.6 Behavioral classifier development

#### 2.6.1 Training data set construction

Triaxial (*x*, *y*, *z*) accelerometer data from three individuals fitted with STCs were used to develop the behavioral classifier (Table 1). Six broad behavioral classes (“Continuous Walk,” “Foraging,” “Resting,” “Running,” “Standing,” and “Other”) were established using the criteria listed in the Appendix S1. Behavioral classes were determined based on a collective knowledge of *Sus scrofa* behavioral repertoires within the Czech Republic are consistent with those reported in other Suidae behavioral classification studies (Dentinger et al., 2022; Erdtmann & Keuling, 2020; Zhang et al., 2022). Behaviors were identified using video records, and corresponding accelerometer profiles were located by matching video timestamps with synced timestamps in the DDMT software. Profiles were then extracted to create behavioral ethograms composed solely of triaxial accelerometer data falling into one of the six behavioral classes. To facilitate future refinement of the classifier, “Foraging,” “Running,” and “Standing” classes were further subdivided to produce three additional, “higher resolution” behavioral categories: “Rooting,” “Trotting,” and “Vigilance,” respectively, resulting in a total of nine behavioral classes. The higher‐resolution behavioral classes were collapsed into their parent classes for initial classifier evaluation.

Each marked behavioral epoch was subdivided into 4‐s nonoverlapping windows to generate baseline observations for classifier training (i.e., entities to be classified following feature extraction). The 4‐s observation window was chosen in consideration of two factors: the shortest‐duration behavior desirable to detect and the minimum acceptable detection latency. In total, there were 13,461 training observations (14.96 hours of marked data), with the following breakdown of observations and training percentage for the six “core” behavioral classes: “Continuous Walk” (1445, 11%), “Foraging” (2601, 19%), “Resting” (6345, 47%), “Running” (1042, 8%), “Standing” (1668, 12%), and “Other” (360, 3%) (Table 2, Table S1). Training data for the “higher resolution” behavioral subclasses are provided in Table 2, Table S1. The proportions of observations used to train the behavioral classifier were selected a priori to reflect the frequencies of these behavioral classes thought to occur in natural contexts (VS, MJ personal observations). The training data set was constructed from three individuals (B3, B6 male; B5, female), all fitted with STCs with identical tag orientations (Figure 1a, Table 1).

**TABLE 2:** Summary of behavioral data used for classifier training. For each behavioral class, the total duration (seconds) and total observations (i.e., 4‐s training windows) are shown, as well as the class proportion (%). A total of 14.96 h of training data were used for classifier development and proportions reflect those of the test set. The expanded suite of “higher resolution” behavioral classes are italicized and nested within the respective parent class (i.e., 669 of the “Forage” observations were subclassified as “Root,” representing 5% of all training observations).

Classifier training: behavioral class summary			
Behavioral class	Total duration (s)	Total observations	%
Rest	25,380	6345	47.1
Forage	10,404	2601	19.3
*Root*	*(2676)*	*(669)*	*(5.0)*
Walk	5780	1445	10.7
Stand	6672	1668	12.4
*Vigilance*	*(472)*	*(118)*	*(0.9)*
Run	4168	1042	7.7
*Trot*	*(2556)*	(639)	*(4.8)*
Other	1440	360	2.7
Total	53,844	13,461	100.0

#### 2.6.2 Feature extraction

Eighteen features were extracted from each 4‐s raw‐data observation window. These features were the estimated “signal power” in each of four frequency bands (0–2.5 Hz, 2.5–5 Hz, 5–7.5 Hz, and 7.5–10 Hz; four features), the signal median (one feature), and the signal variance (one feature), for each of the three accelerometer axes. The power features were derived from the Welch method of power spectral density estimation (2 s windows with 1 s overlap, 64‐point Discrete Fourier Transforms), by integrating the output in the designated frequency ranges. All features guaranteed to be nonnegative (i.e., all except the median features) were log‐transformed to a decibel‐proportional scale prior to further processing. Finally, features were *z*‐scored and principal component analysis was performed, retaining a number of components required to preserve 95% of the total data variance (eight components). The resultant 13,461 × 8 matrix served as the training data for a 5‐nearest neighbor classifier with cityblock distance as the metric (Hastie et al., 2009). A k‐NN classifier was chosen for its ability to represent highly nonlinear decision boundaries, based on the demonstrated success of similar methods in prior biologging studies (Bidder et al., 2014; Painter et al., 2016; Sur et al., 2017). The number of neighbors and distance metric were chosen by grid‐search optimization on a small holdout data set (Hastie et al., 2009).

#### 2.6.3 Behavioral classifier evaluation

Performance of the behavioral classifier was evaluated using continuous accelerometer recordings collected from three individuals (B4, B7, and B30) not used in classifier training (Table 1). Prior to evaluation, behaviors were verified using ground truth video recordings and corresponding accelerometer profiles were identified as described above. Because the tag orientation was not identical across training and test boar (Figure 1, Table 1), test data *x*, *y*, and *z* acceleration vectors at every time step were multiplied by the 3D rotation matrix required to map them to the coordinate frame used for training data. After 18‐dimensional feature extraction, test data observations were transformed using the training data mean and standard deviation vectors before being projected onto the 8‐dimensional principal component space of the training data for classification. Initial test data classifications were made at every possible time step using a 4‐s symmetric, noncausal sliding window.

#### 2.6.4 Postprocessing

Initial classifications were smoothed with a nonlinear filter; specifically, the class at each time step was replaced with the modal class of a 1‐s forward‐looking window. This filtering step resulted in a set of candidate's behavioral events, each delimited by a starting and ending time, which were then subject to two predetermined heuristic criteria to yield the final set of classifications. The first was that each candidate behavioral event was required to be of a minimum duration: “Foraging” (5 s; “Rooting” 3 s), “Resting” (120 s), “Running” (3 s; “Trotting” 3 s), “Standing” (2 s; “Vigilance” 2 s), and “Other” (1 s). Any candidate event not meeting its minimum duration, which was used only in postprocessing, was reassigned to the next most likely class for which the duration criterion could be met. Class likelihoods were determined using the relative class proportions among the five nearest training set‐neighbors corresponding to each time step in the candidate event. Class proportions were summed across time steps and sorted to produce a rank‐ordered likelihood for the classes. Candidate events for which this procedure failed to yield a valid alternate class assignment were merged with the subsequent event.^1^

The second heuristic was that any candidate “Standing” event flanked by “Resting” activity was reassigned to the “Resting” class. Specifically, this reassignment was made if the majority of a 120 s window on either side of the candidate “Standing” event was classified as “Resting.”

### 2.7 Magnetometer data

To assess magnetic compass heading accuracy and reliability, magnetometer data were collected from four collars under two conditions: a controlled laboratory environment (hereafter, “lab evaluation”) designed to test the precision of the magnetometer and from three free‐roaming boar inside the behavioral enclosure (hereafter, “field test”) (Table 1).

During the lab evaluation, the tag was leveled and centered inside an electromagnetic enclosure containing four Helmholtz's coils used to manipulate the strength and alignment of an experimentally generated magnetic field. Two orthogonally aligned coils were used to cancel the residual horizontal component of the Earth's magnetic field (+/− 0.1%) and to adjust the vertical component of the magnetic field to match that of an Earth strength vertical field (~45,000 nT). Two inner orthogonally aligned coils were used to generate Earth‐strength magnetic fields (total strength ~50,000 nT) that could be rotated into alignment into one of four cardinal compass alignments corresponding to topographic North, South, East, and West (Kirschvink, 1992). The tag was oriented such that one end of the *x*‐axis was aligned toward topographic North which was then defined as the “heading direction” in DDMT for analysis. Tag orientation remained static, whereas the horizontal component of the magnetic field was rotated by 90° increments into alignment with each of the four cardinal compass directions for a period of 10 s in each alignment. Magnetic heading measurements calculated by DDMT were plotted relative to the four expected cardinal compass directions using the gghistogram function in the R package ggpubr (Kassambara, 2020).

Field tests of magnetometer performance were carried out concurrently with data collected for the behavioral classifier within the behavioral enclosure on three free‐roaming individuals (Table 1). Video recordings were used to estimate ground truth magnetic headings and a spatial array of “magnetic landmarks” were installed within the camera's field of view to provide known magnetic references to better estimate magnetic headings of focal subjects. Magnetic landmarks were either nonmagnetic cables tethered between trees or the nonmagnetic fence‐line forming the behavioral enclosure (Figure S1). A total of 45 independent behavioral epochs from all core behavioral classes (excluding “other”), totaling 5:27 (min:s), were selected to test the precision of the magnetic heading data. Heading predictions were made by two investigators not involved in data collection and blind to all raw magnetometer data. Using only video records, investigators predicted boar magnetic heading using the available magnetic landmarks described above. For each prediction, the average magnetic heading was estimated over the duration of the behavioral segment identified. When investigator predictions differed by less than 20° (*n* = 40), they were averaged to establish the final magnetic heading, whereas when predictions differed by more than 20° (*n* = 5), investigators determined a final prediction after reevaluating the recording together. A third investigator blind to the magnetic predictions extracted the magnetic heading data from DDMT which was later compared to investigator predictions.

## 3 RESULTS

### 3.1 IMSC field performance: Durability, capacity, and lifetime

Between 2019 and 2022, 67 of the 71 total collars (~94%) deployed on free‐ranging boar were recovered and data recording durations ranged from 9 to 421 days. The remaining four collars (~6%) experienced an unknown GPS malfunction and remain unrecovered. Of the 67 collars retrieved, 51 (76%) were fully functional and no appreciable damage was noted, whereas 11 (16%) exhibited mechanical damage likely due to physical stresses associated with boar behavior, and the remaining 5 collars (7%) failed prematurely due to an unexpected electrical fault. Of the fully functional subset, 35 (69%) collars recorded data until retrieval, whereas 9 collars (18%) recorded data for >50% of the deployment period and the remaining 7 collars (14%) recorded data for <50% of deployment period. Overall, free‐ranging boar equipped with IMSCs were tracked for 6001 days and a total of 4547 days of biologging data were recorded, corresponding to 75% of the cumulative deployment duration.

### 3.2 Behavioral classifier

**FIGURE 2:**
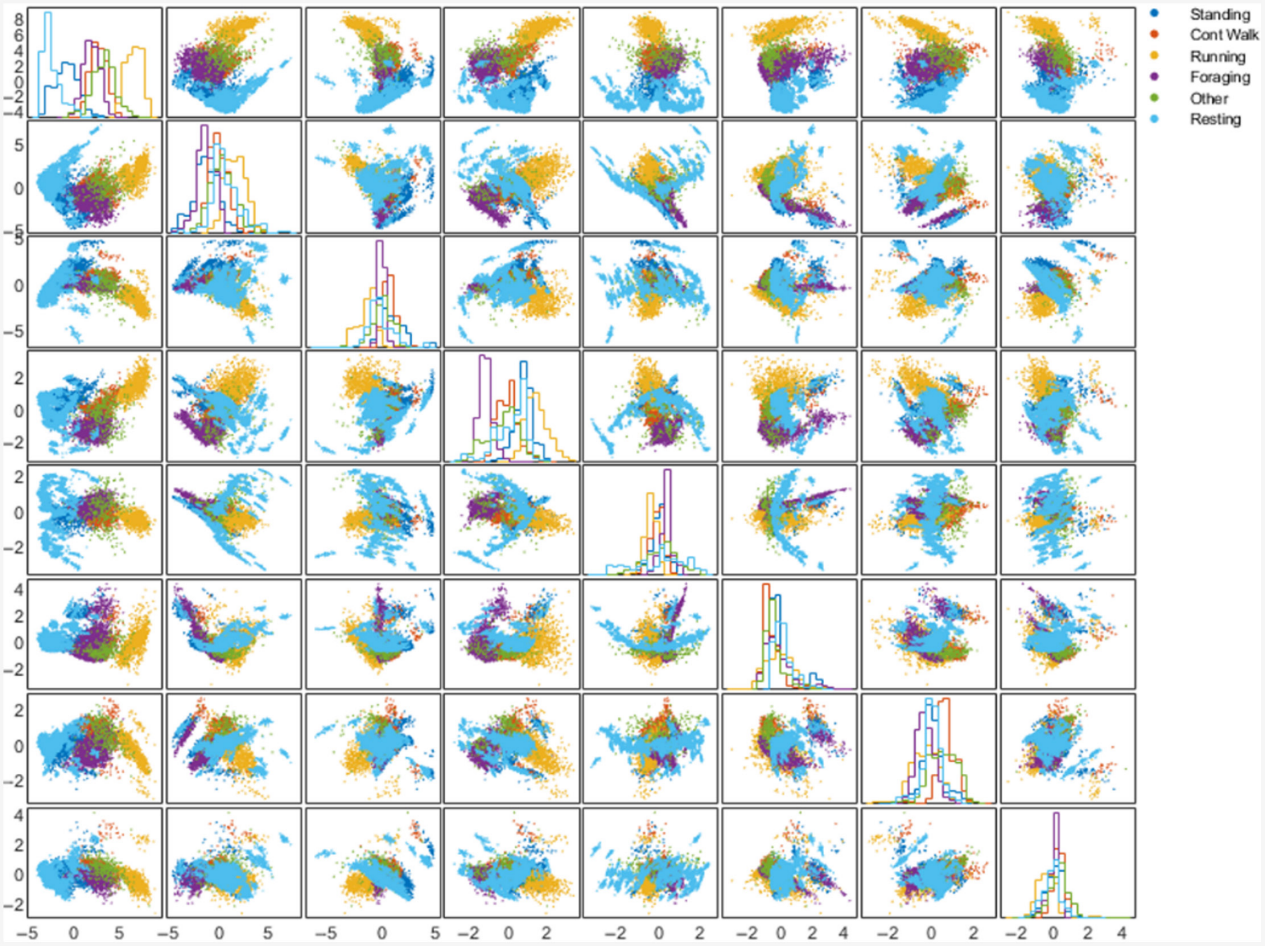
Matrix showing plots of all possible pairs of the 8 principal components (PCs) that were used in behavioral classification. Points correspond to training observations (*n* = 13,461 in each plot) and are colored according to behavioral class. Numbering columns and rows each from 1 to 8, respectively, beginning at the top left corner of the matrix, the column number corresponds to the PC plotted on the horizontal axis and the row number to the PC plotted on the vertical axis. For example, the plot in row 3, column 2 has the second PC plotted on the horizontal axis and the third PC plotted on the vertical axis. The plots along the diagonal are histograms, colored by class, for each of the 8 PCs. (Plots mirrored across the diagonal show the same two PCs with the axes swapped.)

Classifier performance was evaluated using accelerometer data from 2100 independent ground truth behavioral epochs (i.e., independent behaviors falling into one of the six behavioral classes) across three individuals, totaling 08:28:15 (HH:mm:ss) of data (Table 3, Table S1). Classifier performance was evaluated on an event‐by‐event basis (i.e., per 0.1 s sample). Overall behavioral classifier performance was 85.1% across all behaviors from all three individuals (Table 4) and includes data from the STC and IMSC designs with different tag positions and orientations. Of the five behavioral classes of interest (i.e., excluding “Other” which was composed of heterogeneous behaviors only identified by the classifier when a behavior did not fall into any of the five core behavioral categories), the likelihood that any given prediction matched the ground truth class label (i.e., precision), ranged from 77.1% (“Walking” and “Standing”) to 96.5% (“Resting”) (Table 4). Classifier recall, that is, the proportion of behavioral epochs correctly identified by the classifier, ranged from 74.7% (“Running”) to 91.8% (“Resting”) (Table 4). Classifier performance was consistent between the three deployments, ranging from 83.5% (B4) to 89.9% (B30), and surprisingly, the collar with the highest performance (B30, IMSC) was least similar in design (i.e., tag position and orientation) to those used to train the classifier (Table 5). All possible pairs of the eight principal components used to identify the six behavioral classes are plotted, along with histograms corresponding to each component in isolation, to illustrate the collective and relative contribution of the principal components toward class separability (Figure 2). Precision and recall metrics were substantially lower when tested on the three expanded behavioral classes, reflecting their similar acceleration profiles relative to their respective parent classes. However, overall classifier performance remained robust, with an accuracy of 78.4%, although there was larger variation in performance between collar designs when tested on the expanded classes (Table 6).

**TABLE 3:** Classifier testing data. Summary of accelerometer data used to test behavioral classifier performance in three individuals not used for classifier training. The total duration (s) and total number of independent behaviors per class (Epochs) as well as their proportions (%) in classifier testing are listed. The expanded suite of “higher resolution” behavioral classes are italicized and nested within their respective parent class, as in Table 2.

Behavioral classifier testing data				
Behavioral Class	Duration		Epochs	
	Sum (s)	%	Total	%
Rest	11,008	36.1	67	3.2
Forage	7436	24.4	343	16.3
*Rooting*	*(1562)*	*(5.1)*	*(98)*	*(4.7)*
Walk	4129	13.5	459	21.9
Stand	4590	15.1	685	32.6
*Vigilance*	*(439)*	*(1.4)*	*(106)*	*(5.0)*
Run	1827	6.0	157	7.5
*Trot*	*(647)*	*(2.1)*	*(106)*	*(5.0)*
Other	1505	4.9	389	18.5
Total	30,495	100	2100	100

**TABLE 4:** Behavioral classifier performance. Confusion matrix showing behavioral classifier accuracy tested on three individuals across six behavioral classes. Classifier predictions are listed on the left column and ground truth classes are listed across the second‐to‐last row. Values within the matrix represent the total number of events for each predicted class (rows) and for each ground truth observation (columns), where an event corresponds to one acceleration data point recorded by the logger. Light green‐shaded cells inside the matrix represent classifier predictions that match ground truth observations. The likelihood that the classifier prediction matches that of the ground truth observation for each behavior class is represented by the Precision column shown on the right. The proportion of events in each class identified by the classifier is represented by Recall shown across the bottom row. Lighter to darker shades of green in Precision and Recall cells indicate lower to higher classification performance, respectively.

Prediction	Behavioral classifier confusion matrix						Precision (%)
Walk	36,851	2883	0	5514	472	2064	**77.1**
Other	2758	9863	15	5606	1580	3881	**41.6**
Rest	39	253	101,077	0	0	3379	**96.5**
Forage	962	1090	0	62,545	2361	995	**92.0**
Run	167	788	0	35	13,649	173	**92.1**
Stand	508	174	8987	656	209	35,409	**77.1**
**Truth**	Walk	Other	Rest	Forage	Run	Stand	
**Recall (%)**	**89.3**	**65.5**	**91.8**	**84.1**	**74.7**	**77.1**	

**TABLE 5:** Behavioral classifier performance summary. Precision and recall percentages are shown for all six behavioral classes, partitioned by individual, as well as overall classifier accuracy (%) per individual.

Behavioral classifier performance summary						
Behavioral class	B4		B7		B30	
	Precision (%)	Recall (%)	Precision (%)	Recall (%)	Precision (%)	Recall (%)
Walk	77.8	86.4	74.2	91.2	87.1	85.6
Other	34.1	58.0	53.7	72.3	4.3	17.4
Rest	93.5	85.6	98.3	95.0	98.6	100.0
Forage	96.1	88.6	62.9	39.1	94.6	96.2
Run	92.3	78.4	94.1	75.2	75.0	60.2
Stand	58.7	72.4	85.9	83.0	95.3	67.3
Overall accuracy (%)	83.5		**84.2**		**89.9**	

**TABLE 6:** Expanded classifier performance. Confusion matrix showing behavioral classifier accuracy tested on three individuals across the expanded suite of nine behavioral classes. Table format is identical to that shown in Table 4.

Prediction	Behavioral classifier confusion matrix									Precision (%)
Walk	36,851	2883	0	4643	871	172	300	1885	179	**77.1**
Other	2758	9863	15	3959	1647	601	979	3334	547	**41.6**
Rest	39	253	101,077	0	0	0	0	3330	49	**96.5**
Forage	170	587	0	42,458	4415	1	0	457	478	**87.4**
Root	792	503	0	7061	8611	2360	0	55	5	**44.4**
Run	0	129	0	35	0	7164	109	15	0	**96.1**
Trott	167	659	0	0	0	1306	5070	119	39	**68.9**
Stand	434	117	8372	585	64	203	2	27,766	2980	**68.5**
Vigilance	74	57	615	7	0	0	4	4547	116	**2.1**
**Truth**	Walk	Other	Rest	Forage	Root	Run	Trot	Stand	Vigilance	
**Recall (%)**	**89.3**	**65.5**	**91.8**	**72.3**	**55.2**	**60.7**	**78.4**	**66.9**	**2.6**	

### 3.3 Magnetic heading: Lab evaluation

**FIGURE 3:**
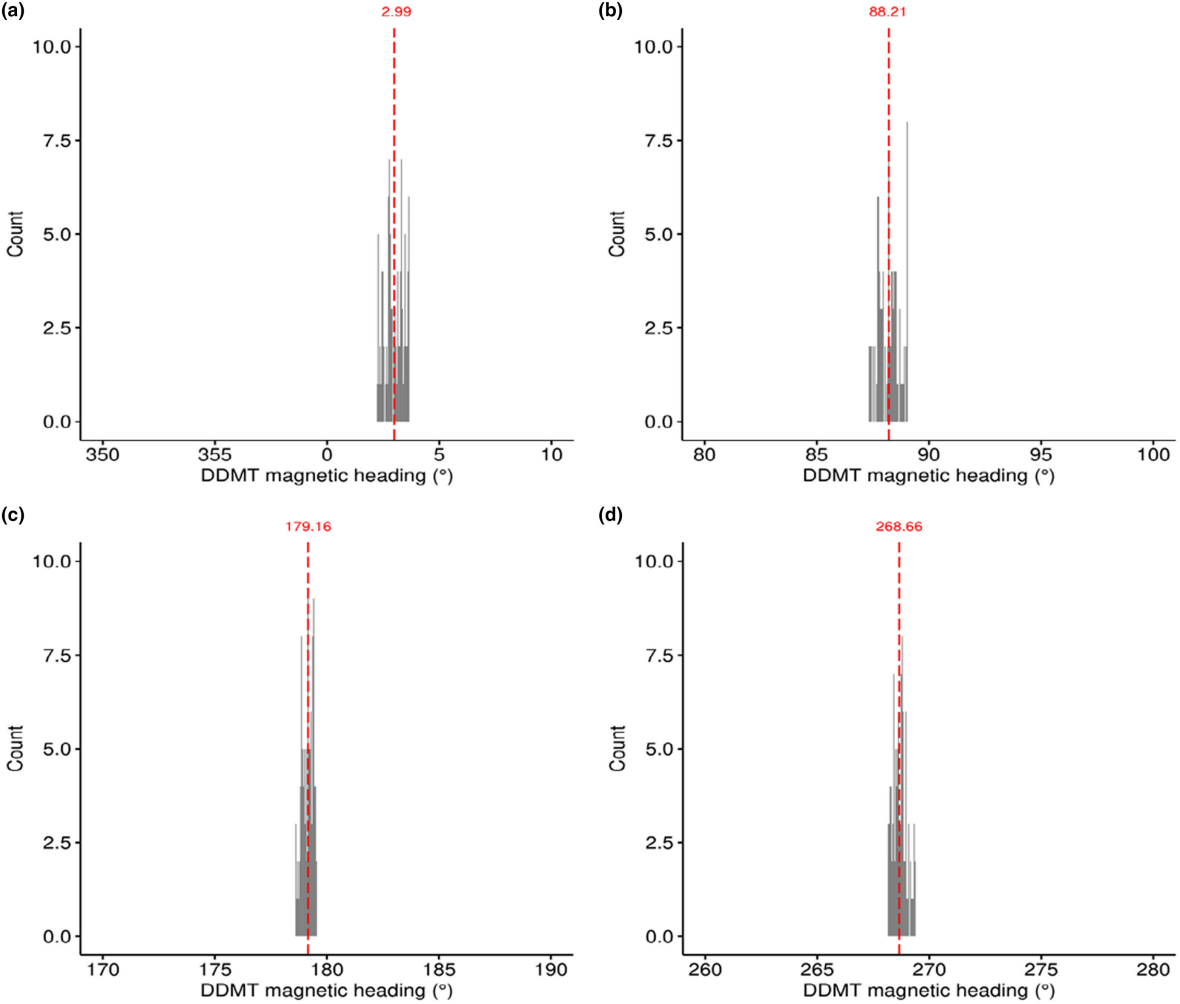
Lab test of triaxial magnetometer data used to calculate magnetic heading measurements after calibration in DDMT software. Histograms plot the total count of 100 samples (10 Hz × 10 s) recorded in each magnetic field alignment (i.e., mN = topoN, E, S, W), relative to magnetic heading bearings calculated in DDMT after performing magnetometer calibration procedures. Plots (a–d) correspond to experimentally generated Earth‐strength magnetic fields aligned at North (0°), East (90°), South (180°), West (270°), respectively. Median values for each magnetic field alignment are shown in red.

Following the calibration procedures described above, the median magnetic heading measurements calculated by DDMT were in agreement with each of the experimentally generated magnetic field alignments: N = 2.99°, S = 179.16°, East = 88.21°, W = 268.66° (Figure 3), with an overall median heading error of 1.7° relative to expected.

### 3.4 Magnetic heading: Field test

**FIGURE 4:**
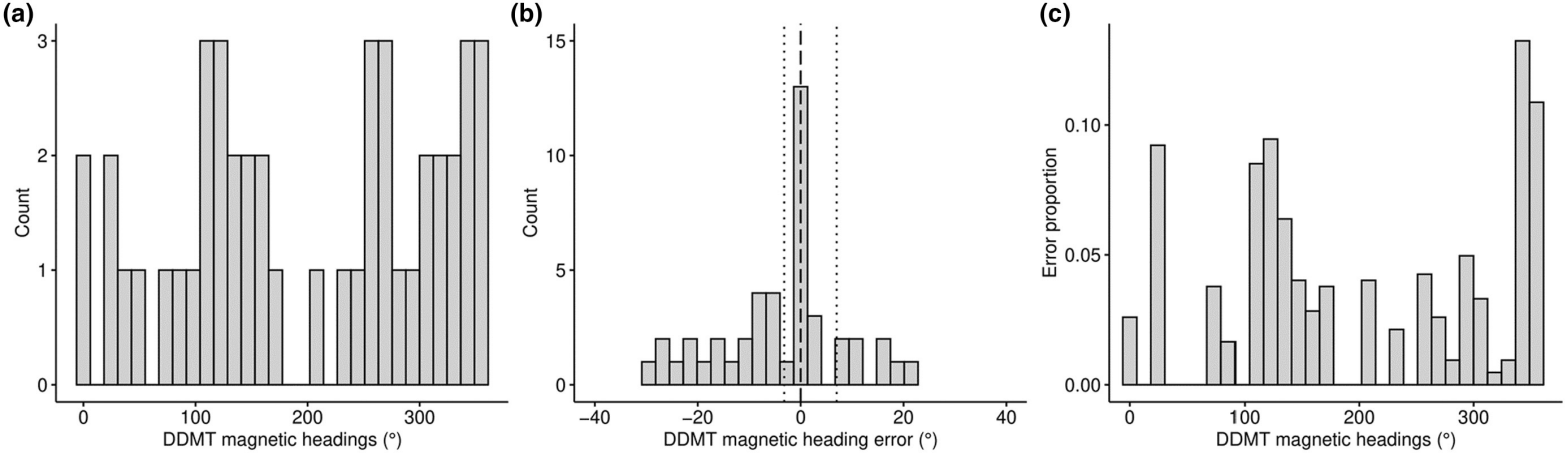
Results from the magnetometer field test collected from free‐roaming individuals equipped with STC and IMSC designs. A total of 45 samples were evaluated and compared to ground‐truth predictions of magnetic heading. (a) Histogram of the overall distribution of magnetic compass measurements produced by DDMT shows that samples were obtained from the range of possible compass directions. (b) The discrepancy between DDMT magnetic compass measurements and ground‐truth recordings, that is, DDMT magnetic heading output error (median error = 0°, black dashed line; bootstrapped 95% CI: −3.1° and 6.9°). (c) The error produced by DDMT was uniform across the range of possible magnetic compass headings. IMSC, integrated multisensor collars; STC, single‐tag collars.

Across all 45 magnetic heading samples, the median discrepancy between DDMT magnetic compass heading measurements and ground truth predictions was 0° (CI: −3.1° and 6.9°) (Figure 4b). Median bootstrapped 95% confidence intervals relative to predictions were calculated using the function boot from the boot package (Canty & Ripley, 2020). Discrepancy between DDMT heading and corresponding ground truth prediction ranged from −30° to 21° (Figure 4b). As shown in Figure 4a, the distribution of compass headings obtained was evenly distributed across all possible magnetic heading alignments, and the error in the DDMT magnetic compass heading measurements compared to predictions was uniform (i.e., error was unbiased across the range of magnetic directions) as indicated by the manova model previously described in Landler et al. (2022) (model results: intercept: approx. *F* = 0.65, *p* = .53, error proportion: approx. *F* = 0.79, *p* = .46) (Figure 4c). The “error proportion” was calculated as the angular deviation between the DDMT measurement and the ground truth prediction divided by the total angular deviation. The cosine and sine of the magnetic heading in radians were used as the response variables and the error proportion as a linear covariate. The intercept of this model was used to test for a significant departure from uniformity (Landler et al., 2022). Importantly, the accuracy of magnetic compass headings was consistent across all three individuals evaluated (Table 7), fitted with different collar designs, biologger positions, and orientations (Figure 1, Table 1), as well as across all behavioral classes, including behaviors characterized by large acceleration amplitudes and variation (e.g., “Foraging,” “Walking,” “Running”).

**TABLE 7:** Magnetic heading field test. Summary and results of ground truth magnetic compass headings. The proportion of time (% Duration) and the proportion of epochs from each behavioral class (% Epochs) used to ground truth the magnetic headings are listed, partitioned by individual. Combined data from all individuals tested, per behavioral class, are shown. Median magnetic heading measurements calculated by DDMT, relative to ground truth predications are shown per behavioral class and partitioned by individual. In cases with a negative (−) median value, the corresponding magnetic compass heading is shown in parentheses.

Behavioral class	B6		B7		B30		Combined		Median heading Rel GT prediction
	% duration	% epochs	% duration	% epochs	% duration	% epochs	Total % duration	Total % epochs	
Rest	0	0	0	0	1	2	1	2	**−8° (352°)**
Forage	30	22	10	9	16	11	55	42	**−6° (354°)**
Walk	1	2	6	9	3	7	10	18	**0°**
Stand	5	7	17	13	11	16	33	36	**−0.5 (359.5°)**
Run	0	0	1	2	0	0	1	2	**9°**
Total	35	31	34	33	31	36	100	100	**n/a**
**Median Heading Rel GT Prediction**	**−0.5° (359.5°)**		**0°**		**−6° (354°)**				**0°**

## 4 DISCUSSION

Animal‐borne telemetry systems have emerged as a powerful tool to further characterize animal movement, behavior, and ecology. The availability of reliable collar systems equipped with a range of sensor technologies adaptable across multiple studies and species is valuable for several reasons, including that it eliminates the need to develop and test novel equipment, and that data sets collected from a standardized system may catalyze additional collaboration, data sharing, and advance progress in analytical techniques.^2^ The IMSC developed here, equipped with triaxial accelerometer and magnetometer sensors, GPS technology, as well as a variety of additional sensors not used in the current study, has proven to be highly reliable under the harsh demands imposed by wild boar under natural contexts. Across the 71 IMSC deployments, 94% of the collars were recovered resulting in biologging data recorded across 75% of the cumulative deployment duration. While the maximum recording duration was an impressive 421 days for one deployment, the majority of IMSCs (72%) were terminated prematurely due to hunting or automobile collisions, which does not reflect collar capacity. In a separate study, 36 IMSCs identical to those described above were deployed on free‐ranging red deer (*Cervus elaphus*) and had an average and maximum data recording duration of 203 and 529 days, respectively. Given the standardization, durability, and functionality of the IMSC, these collars are well suited for long‐term studies in terrestrial mammals, and we hope they will be adopted for use in future biologging studies.

Concurrent with the IMSC development, we have built a behavioral classifier capable of identifying ecologically relevant behaviors from six behavioral classes in wild boar. The classifier had an overall performance of 85% and, of the five core classes (i.e., excluding “Other”), “Resting” was identified with the highest precision, and “Standing” had the lowest precision, most often misclassified as “Resting”, likely due to the similar acceleration profiles between resting and standing behaviors. Classification recall performance was highest in “Resting” and lowest in “Running.” The majority of undetected “Runs” were misclassified as “Forage,” a class that includes “Rooting” characterized by large and variable *x*‐axis acceleration amplitudes, like those associated with “Running” accelerometer profiles. Importantly, the test data set for core and expanded behavioral classes reflected the proportions of behaviors used in classifier training, which in turn, approximated the overall behavioral repertoire of wild boar in natural contexts. This proportionality helps to mitigate performance biases caused by over‐ or underrepresented behaviors and better reflects true overall classification performance. The decision to use a k‐NN classifier was based on classification performance reported in previous biologging literature (Bidder et al., 2014; Painter et al., 2016; Sur et al., 2017), coupled with the nonlinearity of its decision boundaries (Hastie et al., 2009), however, further optimization across classifiers and of hyperparameters could potentially yield performance improvements that can be empirically characterized in future studies.

The classifier exhibited the best overall performance (89.9% accuracy) when tested with data collected from the IMSC, despite being trained on data exclusively from STCs, suggesting that the classifier has an inherent plasticity and is capable of classifying behaviors from biologging tags attached in various orientations and positions. As expected, classifier performance on the expanded suite of behavioral classes was not as robust, largely due to the similarities between the parent class and higher resolution classes. To explore this further, we build upon the framework detailed in Wilson et al. (2018) using DDMT's *Behavior Builder* and *Time Series* functions in an attempt to distinguish between behavioral classes with similar acceleration profiles, such as “Standing” and “Vigilance” behaviors. Applying these postclassification techniques to a subset of our current dataset drastically improved “Vigilance” resulting in >50% precision and recall metrics (see Figure S1). Although encouraging, a more detailed investigation using larger data sets across multiple behaviors will be needed. Furthermore, we expect that a similar improvement in classification performance could also be achieved in the preprocessing stages of classifier development by creating new features that capture subtle differences in accelerometer signatures between similar classes, like those identified between “Standing” and “Vigilance” behaviors.

The classifier was trained and tested solely from triaxial accelerometer data, an important a priori consideration. Because spatial features of the behavioral enclosure remained consistent throughout the study (e.g., location of water source and feeding area, shaded areas used as bedding sites), including locations and viewing angles of the cameras used to collect ground truth videos, it was important to exclude magnetometer data from classifier training and testing, as behaviors under these circumstances cannot be assumed to be randomly oriented. For example, in our study, “Resting” alignment was biased due to limited shaded areas in the enclosure. Had magnetometer data been included in the behavioral analysis, the classifier would likely identify “Resting” using biased magnetometer data that have no relevance beyond the confines of the behavioral enclosure and would result in false positive classifications that artificially inflate precision and recall metrics. We acknowledge that magnetometer data can be valuable for behavioral identification under certain contexts (Chakravarty et al., 2019; Williams et al., 2017); however, it remains unclear if studies that incorporate magnetometer data into machine learning analyses could be predisposed to such biases, as the relative contribution of magnetometer data used for behavioral identification is rarely provided.

Nonetheless, triaxial magnetometer data can provide a wealth of opportunities for exploring movement ecology in greater detail, such as dead‐reckoning analyses (Gunner et al., 2020) and studies of magnetic alignment (Begall et al., 2013; Červený et al., 2017). Given the salience of magnetometer data in biologging research, it is surprising that few studies have validated the precision of magnetic compass headings calculated from raw triaxial magnetometer data (but see Wilson et al., 2007). Therefore, we provide a detailed characterization of magnetic heading measurements under laboratory and natural contexts with magnetometer sensors mounted in different positions and orientations. Magnetic headings calculated by DDMT were consistent with ground truth predictions, with an overall median deviation from expected of 1.7° and 0° in the laboratory and field test, respectively. These data confirm that the magnetometer calibrations (i.e., soft‐ and hard‐iron corrections) and tilt‐compensation algorithms applied in DDMT are well suited for extracting high‐frequency magnetic compass bearings from raw magnetometer data.

Importantly, the field test carried out on free‐roaming boar included magnetic measurements that were obtained from the core behavioral classes. “Running” had the largest average deviation relative to expected (9°) and may be due to the large variation in acceleration amplitudes that introduce “noise” into accelerometer‐dependent tilt‐compensation calculations and/or the (in)ability of observers to accurately predict magnetic alignment from more spatially erratic behavioral classes, such as “Running.” Unintuitively, however, magnetic headings obtained from “Resting” behavior, characterized by little‐to‐no variation in acceleration profile, also had a relatively high deviation from expected (8°) and was likely due to an obstructed view of the animal's head alignment caused by a dense canopy covering the bedding area where boar would exclusively rest. It is noteworthy that magnetic compass performance remained accurate across all core behaviors (excluding “Other”, which was not assessed), and compass performance was evaluated across a representative range of all possible magnetic directions (i.e., 0°–359°). This is the first study to our knowledge that has provided a detailed characterization of magnetic compass performance in free‐roaming animals using ground truth data.

**FIGURE 5:**
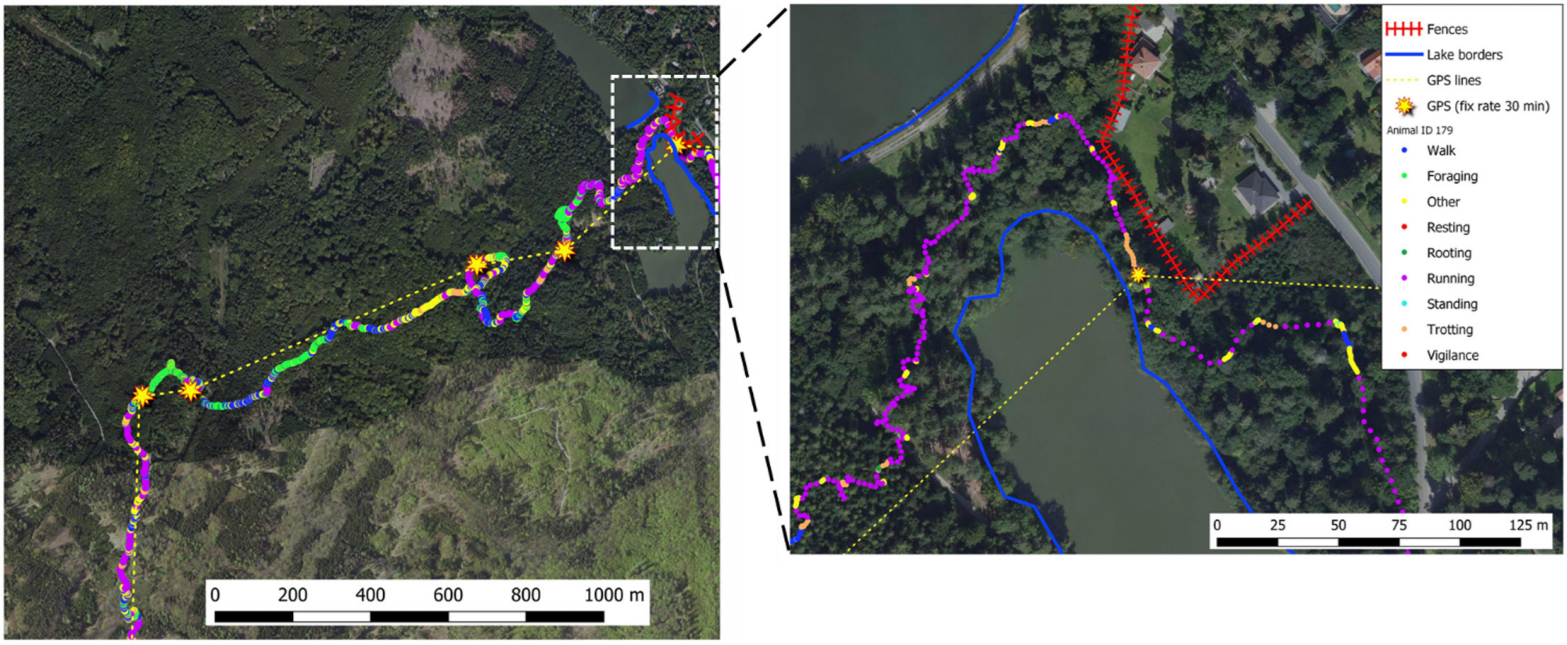
Example of dead‐reckoning path reconstruction in a free‐ranging wild boar equipped with the IMSC. Behaviors were first identified from continuous accelerometer data using the behavioral classifier and were then uploaded to DDMT. User‐defined speed coefficients were assigned to each behavioral class which was then integrated with time‐synced magnetic heading data to reconstruct movement traces between GPS fixes, which anchored the tack to the landscape. Right inset shows a detailed track segment that avoids obvious environemental boundaries and physical barriers, highlighting the added value of dead‐reckoning analyses compared to using GPS data alone and lends further credibility to the precision of the dead‐reckoning methodology used in this study.

Of particular interest is the implementation of dead‐reckoning to reconstruct high‐resolution movement traces in free‐roaming mammals. As a proof‐of‐concept, we take advantage of three important elements made possible by the IMSC presented in the current study: (i) the behavioral classifier capable of identifying ecologically relevant behaviors in free‐roaming boar, (ii) a reliable stream of magnetic heading data recorded at subsecond intervals, and (iii) GPS fixes recorded at 30‐min intervals. Dead‐reckoning relies on vector integration, where vectors depend on speed (or distance traveled) and heading estimates derived from raw biologging data (for details, see Bidder et al., 2015; Gunner et al., 2021). Deriving speed from biologging data is notoriously difficult (Cade et al., 2018), and previous work has assigned speed coefficients to manually labeled behavioral classes to estimate vector lengths for dead‐reckoning path reconstruction (Bidder et al., 2015). We build upon this approach by using machine learning to identify behavioral classes from large volumes of continuous biologging data, which were then assigned speed coefficients based on ground truth observations. Coupling our behavioral classification techniques with the accuracy of our verified magnetic heading data yielded high‐resolution track reconstruction that was further refined by “anchoring” tracks to the landscape using time‐synced GPS fixes (Figure 5). The tortuosity of the reconstructed track in Figure 5 that explicitly avoids environmental boundaries and physical obstacles highlights the precision of these methods compared to using GPS data alone and offers a powerful approach to investigate movement ecology over multiple spatiotemporal scales.

Although the emergence of biologging techniques has revolutionized studies of animal ecology, a growing set of challenges accompanies these technologies, requiring multidisciplinary expertise. The IMSCs developed here, coupled with a robust behavioral classifier and a detailed verification of magnetic heading performance, provide a commercially available system that can be adopted and adapted for future studies on terrestrial mammals.

## AUTHOR CONTRIBUTIONS

**Michael S. Painter:** Conceptualization (lead); data curation (lead); formal analysis (equal); investigation (lead); methodology (lead); project administration (equal); validation (equal); visualization (equal); writing – original draft (lead); writing – review and editing (lead). **Václav Silovský:** Conceptualization (lead); data curation (lead); formal analysis (equal); investigation (lead); methodology (lead); project administration (equal); validation (equal); visualization (lead); writing – original draft (lead); writing – review and editing (lead). **Justin Blanco:** Conceptualization (equal); data curation (equal); formal analysis (lead); investigation (equal); methodology (equal); software (equal); validation (lead); writing – original draft (supporting); writing – review and editing (equal). **Mark Holton:** Methodology (equal); software (equal); visualization (equal); writing – review and editing (equal). **Monika Faltusová:** Formal analysis (supporting); methodology (supporting); writing – review and editing (supporting). **Rory Wilson:** Conceptualization (supporting); methodology (equal); writing – original draft (supporting); writing – review and editing (equal). **Luca Börger:** Conceptualization (supporting); methodology (supporting); writing – review and editing (equal). **Liza Psotta:** Conceptualization (supporting); data curation (equal); formal analysis (supporting); methodology (supporting); validation (supporting); writing – review and editing (equal). **Fabian Ramos‐Almodovar:** Formal analysis (supporting); methodology (supporting); software (supporting); validation (supporting); writing – review and editing (supporting). **Luis Estrada:** Data curation (supporting); methodology (supporting); software (supporting); writing – review and editing (supporting). **Lukas Landler:** Conceptualization (supporting); data curation (supporting); formal analysis (equal); methodology (supporting); validation (equal); visualization (supporting); writing – original draft (supporting); writing – review and editing (equal). **Pascal Malkemper:** Conceptualization (supporting); funding acquisition (supporting); methodology (supporting); writing – review and editing (supporting). **Vlastimil Hart:** Conceptualization (equal); data curation (equal); formal analysis (supporting); methodology (equal); project administration (equal); validation (equal); writing – review and editing (supporting). **Miloš Ježek:** Conceptualization (equal); data curation (equal); formal analysis (supporting); funding acquisition (equal); methodology (equal); project administration (equal); supervision (equal); writing – original draft (supporting); writing – review and editing (equal).

## FUNDING INFORMATION

This study was supported by “EVA4.0” (grant no. CZ.02.1.01/0.0/0.0/16_019/0000803), OP RDE, and “NAZV” (grant no. QK1910462) and financed by the Ministry of Agriculture of the Czech Republic.

## CONFLICT OF INTEREST STATEMENT

The authors have no conflicts of interest to declare.

## Supporting information


Appendix S1

## Data Availability

All raw triaxial accelerometer and magnetometer data used for behavioral classifier development and tests of magnetic heading performance are available on Dryad at: https://datadryad.org/stash/share/4UhVGcozs_QpPViXRuwXSMTYllzqX3RXpHPWPZA6m1M.

## Endnotes

^1^
If no subsequent event was available – only possible at the end of a file – the candidate event was discarded as unclassifiable. A small number of time steps at the beginning of each file were similarly discarded, since a fixed amount of time must accumulate before the classifier can make its first decision. Such edge‐effects have negligible impact on classifier evaluation.

^2^
We do not imply that the field of animal‐borne telemetry and biologging is not collaborative, and indeed, would argue the opposite. However, we suggest that increased overlap between methodologies may encourage further collaboration and promote the growth of this emerging discipline.
