# Supplementary material for: Development of a multisensor biologging collar and analytical techniques to describe high‐resolution spatial behavior in free‐ranging terrestrial mammals
Source: Ecol Evol. 2024 Sep 23;14(9):e70264. doi: 10.1002/ece3.70264 (PMC11420106; doi:10.1002/ece3.70264)
Supplement: Supplementary file 1 — Appendix S1 [file ECE3-14-e70264-s001.zip › BoarMS_Supplementary Materials_Final.docx]

**Supplementary Materials**

*Study site for behavioral classifier development and magnetometer assessment*

The wildlife reserve housed populations of wild boar (*Sus scrofa*) (pop size = 57 individuals), fallow deer (*Dama dama)* (population size = 12 individuals) and sika deer (*Cervus nippon nippon)* (pop size = 53) free roaming within a ~100-hectare area composed of undisturbed forests, grasslands, freshwater ponds, and small streams. Boar were baited to a common feeding area supplied with corn, carrots, and other feeds. An air rifle (Pnue-Dart G2 X-Caliber) equipped with a tranquilizer dart (Pnue-Dart 3CC Type U RDD) containing 250 mg of Xylazine, 150 mg of Ketamine and 250 mg of Zoletile (dosage based on 90 kg body-mass) was used to sedate and transport individuals to the behavioral enclosure where collars were then securely fastened to the animal using non-magnetic stainless-steel hardware. Boar were allowed to wake naturally without injection of reversal sedative medications.

The environment within the behavioral enclosure closely resembled that of the natural environment of the surrounding wildlife preserve. Several mature pines and deciduous trees were located within the behavioral enclosure, providing shade and bedding sites for the boar, and ground cover was mixed and varied by season (e.g., dirt, low growing grasses, dense shrubs). Because there was no consistent water source within the enclosure, a water trough was provided and feed (e.g., corn and root vegetables) was distributed within a ‘feeding area’ inside the enclosure, both supplied ad libitum. No behavioral data was collected for classifier training or testing when subjects were within 1 m of the water trough to avoid any potential behavioral biases. Game cameras were installed throughout the enclosure used to ground truth accelerometer (i.e., behavioral) and magnetometer (i.e., magnetic heading) data (Fig S1). Upon motion detection, cameras were programmed to record for 60 sec followed by a 30 sec ‘time out’ period to help preserve battery life. Videos were saved to removable SD cards and transferred to a PC for later analysis. Boar were held within the behavioral enclosure for different durations ranging from 4 to 10 weeks throughout the study period. Prior to release, individuals were sedated using identical tranquilization procedures to those described above, at which point collars were manually removed and boar were translocated to a shaded area outside of the enclosure and allowed to wake naturally.

*Single Tag Collar (STC) Specifications*

Combined battery and tag weight totalled 26 g without the protective housing, which consisted of a 12 cm x 4.8 cm dia PVC-U cylindrical tube. Prior to capping the tube ends with KGM socket stoppers, the tube was completely filled with Guronic casting resin, which secured the electronics within the housing, fixed the relationship between the tag and battery, a requisite for the magnetometer sensor axes calibrations, and provided waterproofing and protection from mechanical shock. Housings were then secured to plastic belts using adjustable stainless steel hose clamps or plastic cable ties and were precisely oriented such that the accelerometer and magnetometer axes were fixed in a consistent alignment relative to the frame of the boar for four STC deployments, while the sensor axes in an additional STC deployment was rotated to test classification plasticity (Fig 1, Table 1).

During deployment, collar belts were cut to appropriate size and securely fastened with stainless-steel bolts and locknuts. Housings were positioned on the outside of the belt and centered at the base of the neck for all deployments (Fig 1). Total weight of the STC was 250 g. Before collaring, the Daily Diary was activated using a magnetic reed switch and logged continuously (10 Hz) until the collars were recovered or the battery voltage dropped below 3.0 V, which automatically triggered the tag to power down and preserve the stored data. Upon recovery, housings were opened, the Guronic resin was removed from the tag, and the microSD card was recovered for data analysis.

*Integrated Multi-sensor Collars (IMSC)*

In collaboration with Wildbyte Technologies and Vectronic Aerospace GmbH, the IMSC was developed with the intent of producing a more dynamic, robust, and standardized collar with increased functionality and reliability. Collars were fastened to the individual using non-magnetic stainless steel bolts and locknuts. As with the STCs, the biologger and GPS were powered before deployment and logged continuously until the collars were recovered, the battery voltage dropped below 3.0 V, or the 32 GB SD card was at capacity, at which point only the GPS remained active. Collar devices contained a SIM card enabling automatic upload of GPS data to satellite; if data transmission was not possible, GPS fixes were stored until a reliable connection was established. Initiation of the drop-off feature, allowing the collar to be remotely released from the animal’s neck, triggered the collar to broadcast a VHF signal used to pinpoint the collar during recovery efforts. Once recovered from the field, collars were sent to Vectronic Aerospace where the housings were opened, and data were accessed and uploaded to a shared drive for analysis.

The IMSC was equipped with additional logger sensors (e.g., pressure, temperature, light sensors), which were deactivated to preserve tag lifetime and reduce data volume.

*Tag Orientations*

The position of the logger for all STC deployments was the same, located at the base of the animal’s neck. For B3, B5, B6, and B7, the alignment of the sensor axes is as follows: x-axes aligned parallel to the spine (+ polarity towards posterior end); y-axis aligned perpendicular to the spine (+ polarity towards animal’s left side); z-axis aligned along the animal’s dorsal-ventral axis (+ polarity pointing towards dorsal end) (Fig 1A). To test the plasticity and adaptability of the behavioral classifier, the orientation of the biologging tag deployed on B4 was rotated such that the polarities of the y- and z-axes were oriented precisely 180° relative to the tag orientation described for B3, B5, B6, and B7 (Fig 1B).

For the IMSC deployment, the tag was positioned on the left side of the animal’s neck and the sensor axes were aligned as follows: x-axes aligned parallel to the spine (+ polarity towards posterior end); y-axis aligned along the animal’s dorsal-ventral axis (+ polarity pointing towards dorsal end); z-axis aligned perpendicular to the spine (+ polarity towards animal’s right side) (Fig 1C).

*Behavior Classification Criteria*

Resting: subject laying down on the ventral or lateral side. Head may be resting on ground or raised. Overall, body and head are relatively motionless (minimum = **120** sec)

Foraging: standing, head and snout pointed downward and touching ground. Clear indication that the snout is making contact with ground and subject is sniffing, grasping feed with snout, and/or chewing feed (minimum = **5** sec)

Rooting: similar body position to foraging with the additional movement of forcefully digging/clearing dirt with snout causing a front-to-back swinging motion of the head (minimum = **3** sec)

Continuous walk: uninterrupted walking motion in a consistent trajectory, no more than 1 hoof off the ground at a time during the gait (minimum = **4** sec)

Running: subject in a full sprint resulting in paired hooves (i.e., front hooves or back hooves) off the ground simultaneously (minimum = **3** sec)

Trotting: continuous and rhythmic gait with 2 opposing hooves on opposite sides of the body off the ground simultaneously (e.g., front left and right back hooves off ground) (minimum = **3** sec)

Standing: subject standing motionless, head direction is parallel with spine direction and little to no side-to-side head movement (minimum = **2** sec)

Vigilance: subject abruptly interrupts current behavior, stands still and distinctly raises head to visually search/survey surroundings. Often associated with sniffing and intentionally moves head side-to-side as the individual visually scans surroundings (minimum = **2** sec)

Other: any remaining behavior or movements that do not fall into the categories defined above, including self-scratching, rubbing against trees or other structures, shaking head/body, aggressive behavior towards other subjects, and/or startling disturbances in the environment (minimum = **1** sec)

**Supplemental Figures and Tables**

**
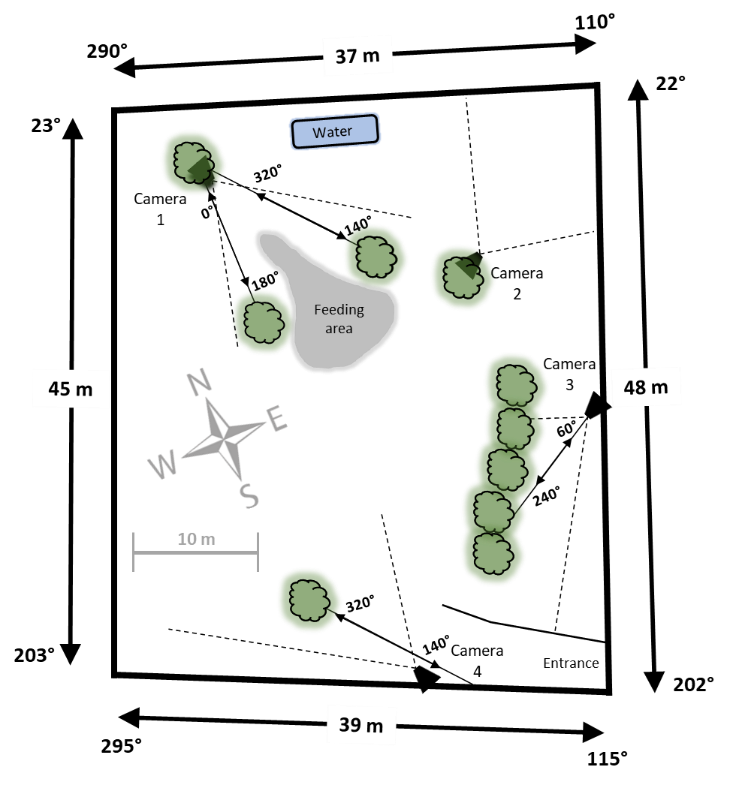
**

**Figure S1.** Schematic of behavioral enclosure used to train and test the behavioral classifier and perform the field test of magnetic compass headings. A total of six boar were collared and released inside the behavioral enclosure equipped with a series of IR motion sensor game cameras (black trapezoids). The approximate field of view of each camera is shown by black dashed lines. Video records were used to create behavioral ethograms that served as training data for the behavioral classifier and provided a ground truth record of behaviors when testing the performance of the classifier. Solid black lines with double-headed arrows and opposing magnetic compass directions represent ‘magnetic landmarks’ secured between trees (green circular objects). The dimensions and magnetic alignments of the enclosure’s perimeters are also shown. Feeding and water areas, as well as a compass rose indicating the cardinal compass directions, are shown.


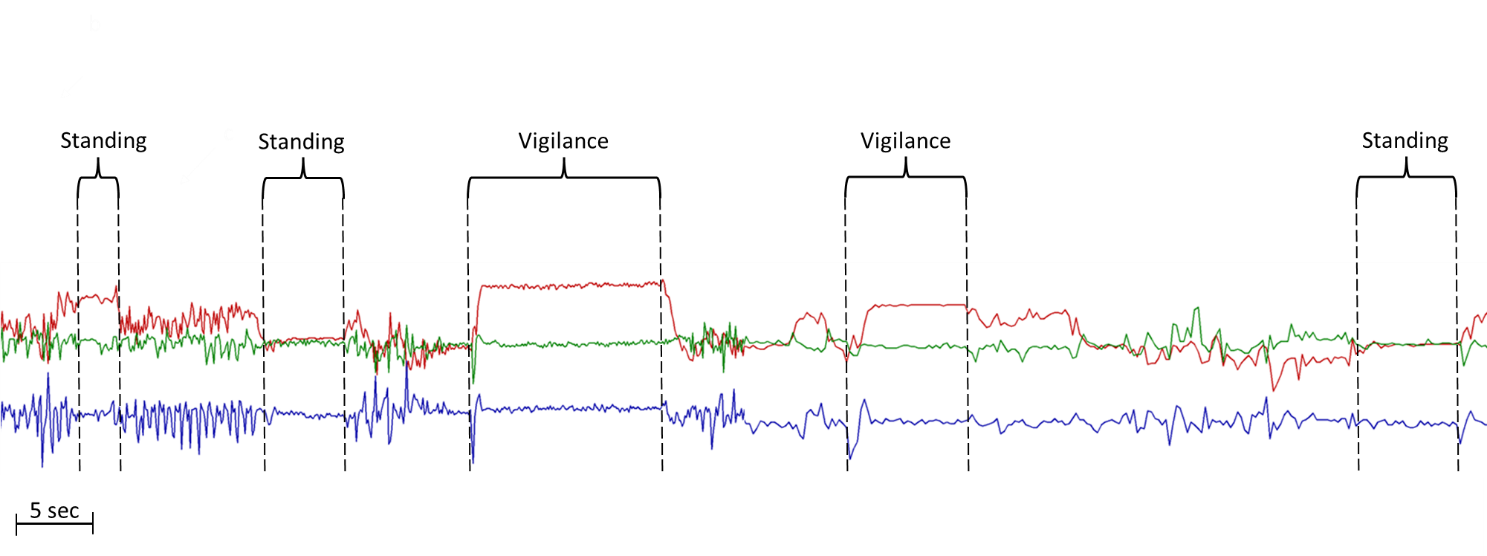


**Fig S2. Example of using DDMT, post-processing to further refine behavioral classification.** Using DDMT post-machine learning behavioral classification may help to distinguish between different behavioral classes with similar acceleration profiles. An example comes for distinguishing between ‘Standing’ (parent class) and ‘Vigilance’ (daughter class) behaviors. Raw acceleration axes recorded from B7 at 10 Hz are as follows: red = x-axis, green = y-axis, blue = z-axis. Acceleration profiles corresponding to ground truth behaviors belonging to each class are identified by black brackets with vertical dashed lines. Although the behavioral classifier did not reliably distinguish the daughter from parent class (i.e., ‘Vigilance’ from ‘Standing’), one possibility is to use the *Behavior Builder* and *Time Series* features in DDMT, post-behavioral classifier. For example, in this study ‘Vigilance’ was defined as a two-part behavior, starting with an abrupt head lift followed by motionless standing or visual scanning behavior and can be visually identified in the distinct change in x-axis acceleration as shown in both ‘Vigilance’ behaviors above. In contrast, ‘Standing’ was defined as a motionless behavior with the snout aligned roughly parallel to the horizontal, and importantly, an initial abrupt head lift was *not* requisite (see corresponding x-axis acceleration profiles during ‘Standing’ behaviors shown above). Therefore, using the *Behavior Builder* feature in DDMT, it is possible to apply an extra layer of classification parameters to previously classified behaviors that, for example, can identify the abrupt change in the x-axis associated with ‘Vigilance’ behavior (an alternative is to use acceleration differentials (not shown) that in some contexts may be more appropriate, as in Wilson et al., 2018. Even though the initiation of ‘Standing’ behavior can be associated with acceleration changes along the x-axis, these changes are often opposite in polarity or are exhibited a slower rate of change (i.e., the head lifting is much less rapid) compared to those of ‘Vigilance’ behavior, a provides a user-defined approach to distinguish between the two similar behavioral classes. See (Wilson et al., 2018) for more details regarding *Behavior Builder* and *Time Series* featuresin DDMT. However, it should be noted that a similar improvement in classification performance between parent and daughter classes could likely be achieved in pre-processing stages of classifier development by creating additional features that specifically capture subtle differences (e.g., an abrupt head lift) between similar behavioral classes.

**Table S1. Classifier Training Data.** Behavioral classifier training data collected from B3, B5, and B6. For each individual, the number of observations (i.e., 4-second training windows) per behavioral class and proportion of each class used in classifier training are shown. The expanded suite of ‘higher resolution’ behavioral classes are also shown (grey rows) and corresponding data is nested within the respective parent class.

**Table S2. Classifier Testing Data.** Data used to evaluate behavioral classification performance, portioned by behavioral class and by individual. For each class, duration (s), and number of epochs (i.e., number of independent behaviors identified from ground truth records) are shown, as well as their proportion in the overall test set. The expanded suite of ‘higher resolution’ behavioral classes are also shown (grey rows) and corresponding data is nested within the respective parent class.

**References**

Wilson, R., Holton, M., Virgilio, A. di, Williams, H., Shepard, E., Lambertucci, S., Quintana, F., Sala, J. E., Balaji, B., & Lee, E. S. (2018). Give the machine a hand: A Boolean time-based decision-tree template for rapidly finding animal behaviours in multisensor data. *Methods in Ecology and Evolution*, *9*(11), 2206.
